# Ultrasound gray scale ratio for differential diagnosis of papillary thyroid microcarcinoma from benign micronodule in patients with Hashimoto’s thyroiditis

**DOI:** 10.1186/s12902-022-01028-0

**Published:** 2022-07-22

**Authors:** Zhijiang Han, Lesi Xie, Peiying Wei, Zhikai Lei, Zhongxiang Ding, Ming Zhang

**Affiliations:** 1 Department of Medical Imaging, The First Affiliated Hospital of Xi’an Jiaotong University, No. 277, Yanta West Road, Xi’an, 710061 China; 2 Department of Radiology, Affiliated Hangzhou First People’s Hospital, Zhejiang University School of Medicine, Hangzhou, China; 3 Department of Pathology, Affiliated Hangzhou First People’s Hospital, Zhejiang University School of Medicine, Hangzhou, China; 4 Department of Ultrasound, Affiliated Hangzhou First People’s Hospital, Zhejiang University School of Medicine, Hangzhou, China; 5 Department of Radiology, Key Laboratory of Clinical Cancer Pharmacology and Toxicology Research of Zhejiang Province, Affiliated Hangzhou First People’s Hospital, Zhejiang University School of Medicine, No. 261, Huansha Road, Hangzhou, 310006 China

**Keywords:** ultrasound gray scale ratio, Papillary thyroid microcarcinoma, Thyroid nodule, Echogenicity, Hashimoto’s thyroiditis

## Abstract

**Background:**

To investigate the diagnostic value of ultrasound gray scale ratio (UGSR) in differentiating papillary thyroid microcarcinomas (PTMCs) from benign micronodules (BMNs) in patients with Hashimoto’s thyroiditis (HT).

**Methods:**

The ultrasound images of 285 PTMCs (from 247 patients) and 173 BMNs (from 140 patients) in the HT group, as well as 461 PTMCs (from 417 patients) and 234 BMNs (from 197 patients) in the non-HT group were retrospectively analyzed. The diagnosis of all cases was confirmed by histopathological examinations. The gray scale values of the nodules and surrounding thyroid tissues were measured and subsequently the UGSRs were calculated. Receiver operating characteristic curve analysis was used to determine the area under the curve (AUC), optimal UGSR threshold, sensitivity and specificity in differentiating PTMCs and BMNs in the two groups.

**Results:**

The UGSR of PTMC and BMN was 0.52 ± 0.12 and 0.85 ± 0.24 in the HT group (*P* < 0.001), and 0.57 ± 0.13 and 0.87 ± 0.20 in the non-HT group (*P* < 0.001), respectively. The difference in PTMC-UGSR was significant between the two groups (*P* < 0.001), whereas BMN-UGSR did not differ between the two groups (*P* = 0.416). The AUC, optimal UGSR threshold, sensitivity and specificity of UGSR for differentiating PTMC and BMN in the HT and non-HT group were 0.890 versus 0.901, 0.68 versus 0.72, 91.23% versus 90.67%, and 77.46% versus 82.05%, respectively.

**Conclusions:**

The USGR of the HT group was lower than that of the non-HT group.

Moreover, UGSR exhibited important diagnostic value in differentiating PTMC from BMN in both HT and non-HT groups.

## Background

The echogenicity of neck strap muscles and thyroid gland are generally used as a reference for evaluating the echo intensity of thyroid nodules. According to the subjective recognition of the observer’s naked eye, the echogenicity is classified into 4 grades as follows: extreme hypoechogenicity (lower than neck strap muscle echogenicity), hypoechogenicity (lower than thyroid gland but higher than neck strap muscle echogenicity), isoechogenicity (equal to thyroid gland echogenicity) and hyperechogenicity (higher than thyroid gland echogenicity). If anechogenicity is included, 5 grades of echo intensity can also be considered. The diagnostic value of hypoechogenicity and extreme hypoechogenicity for malignant tumors have been widely acknowledged [1–4]. However, the conventional echogenicity 4–5 grading method has several disadvantages. Firstly, echogenicity heterogeneity cannot be fully reflected. For instance, all hypoechoic intensities are empowered with the same diagnostic efficiency for the differentiation of malignant from benign nodules, regardless of the difference between these intensities. However, the echogenicity of each nodule just represents one point of a continuous variable, and different points theoretically correspond to different diagnostic efficiencies. Secondly, taking the neck strap muscle echogenicity as a reference has great instability, which is susceptible to various factors, such as the strap muscle thickness evenness, and the proportion of muscle fibers, tendons and adipose tissues. Thirdly, subjective differences in the judgment of echo intensity among observers are inevitable [5–8]. Consequently, choosing a more stable reference to quantify the echogenicity thyroid nodule is of important significance for improving the differential diagnosis of malignant and benign nodules.

When selecting a reference for judging the echogenicity of thyroid nodules, the surrounding thyroid tissue can be considered because it has the following advantages: 1) the location is adjacent to the nodule, resulting in a more intuitive comparison of the images; and 2) the gain and dynamic changes of the nodule echogenicity are accompanied by the corresponding changes in thyroid tissues with the same gain levels, whereas the ratio of the echogenicity is relatively stable. Since 2015, the quantitative studies on the echo intensity of thyroid nodules have all used the surrounding thyroid tissue as a reference. The echogenicity of the nodule has been determined by the ultrasound gray scale ratio (UGSR), although only 5 articles regarding UGSR have been published to date [6–10]. Of these, only patients with HT were included in the study by Grani et al. [6], however, they did not compare the UGSR of benign and malignant nodules in patients with HT alone.

The pathogenesis of HT involves the infiltration of lymphocytes and plasmacytes, the formation of lymphoid follicles in the germinal center, the parenchymal atrophy of thyroid tissues, and the fibrosis of the thyroid gland to varying degrees, resulting in changes of echo intensity of thyroid tissues [11, 12]. At present, no convincing studies have been reported on whether the echo intensity of nodules in patients with HT changes. We speculated that there might be a certain degree of lymphocyte infiltration in and around the nodules, resulting in reduced nodule echogenicity. However, if the echo intensities of nodules, thyroid parenchyma and muscle simultaneously decrease or increase in patients with HT, or one of them slightly decreases or increases, the nodule echogenicity grading based on the traditional 4–5 point classification will not change. In addition, subjective difference in echogenicity judgment between observers should not be underestimated when patients present with HT [13]. Therefore, whether there is a difference in nodule echogenicity between HT and non-HT patients needs to be confirmed by quantification analysis.

In the present study, UGSR was used to quantitatively analyze and compare the echogenicity of PTMC and BMN in the HT and non-HT groups. The finding may help to determine the diagnostic value of UGSR in differentiating PTMC from BMN in HT patients and provide potential evidence for modifying TIRADS.

## Methods

### Subjects

**Fig. 1:**
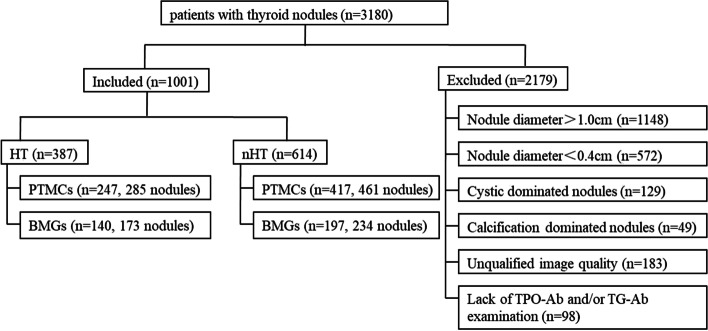
Flow chart of study participant selection

The present study was performed in accordance with the ethics guidelines of the Helsinki Declaration and approved by the ethics committee of our institute. We identified a total of 3,180 consecutive patients with thyroid nodules who were treated in our institution from January 2018 to January 2021. The diagnosis of all cases was confirmed by histopathological examinations. The following nodules were excluded from the analysis: 1) maximal diameter > 1.0 or < 0.4 cm; 2) cystic-dominated nodules (the cystic component was > 50%) [14, 15]; 3) calcification-dominated nodules affecting the measurement of surrounding lesion tissues [7]; 4) lack of TPO-Ab and/or TG-Ab examination; and 5) unqualified image quality. Finally, 1,001 patients with 1,153 thyroid nodules who met the inclusion criteria were included in the study. Figure 1 indicates a flow chart demonstrating the characteristics of the study participants.

### Ultrasonic examination

The following five ultrasonic scanners were used in the present study: Esaote MyLab 70 XVG (Genova, Italy), Esaote MyLab Classic C (Genova, Italy), Esaote Mylab 90 (Genova, Italy), mindray (Shenzhen, China) and Hitachi (Tokyo, Japan). The 5–10 MHz broadband linear array probes were used and the central frequency was 7.5 MHz. The positions and scanning areas of all patients were the same during the scanning process. In brief, the patients were placed in the supine position with the neck dorsal stretched as much as possible to sufficiently expose the anterior neck region. Scanning was performed on the transverse, longitudinal and oblique sections, and the nodule data, including number, size, shape, boundary, halo around the boundary, internal echogenicity, calcification, internal and peripheral blood flow, and bilateral neck lymph nodes, were recorded.

### Measurement of TPO-Ab and TG-Ab

Chemiluminescence immunoassay was performed to measure TPO-Ab and TG-Ab using the Siemens ADVIA Centaur XP automatic chemiluminescence immunoanalyzer (Siemens Medical Diagnosis Ltd, USA). The reference range of the antibodies was ≤ 60ku/L.

### Pathology examination

The tissue samples were processed into slices with an average thickness of 5 μm, which were fixed in 10% neutral formalin, stained by hematoxylin/eosin (H&E), and examined under a light microscopy. All nodules were confirmed by senior pathologists.

### Image analysis

**Fig. 2:**
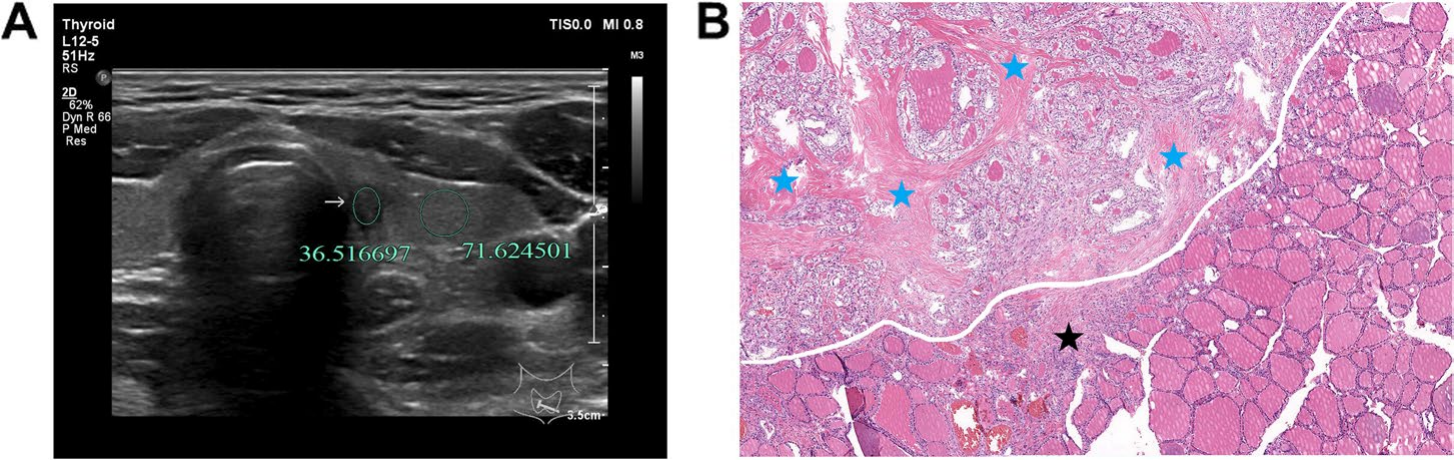
A 39-year-old man with PTMC in the left lobe of the thyroid. **A** the ultrasound transverse image shows that UGSR is 0.51 (36.52/71.62). **B** the pathological image (H&E × 40 magnification) illustrates that the PTMC is located in the left upper part of the image, with high amounts of fiber and collagen hyperplasia (blue stars) inside the lesion, and almost no lymphocyte infiltration. The lesion and surrounding normal thyroid tissues are separated by fibers and minimal lymphocyte infiltration (black star)

**Fig. 3:**
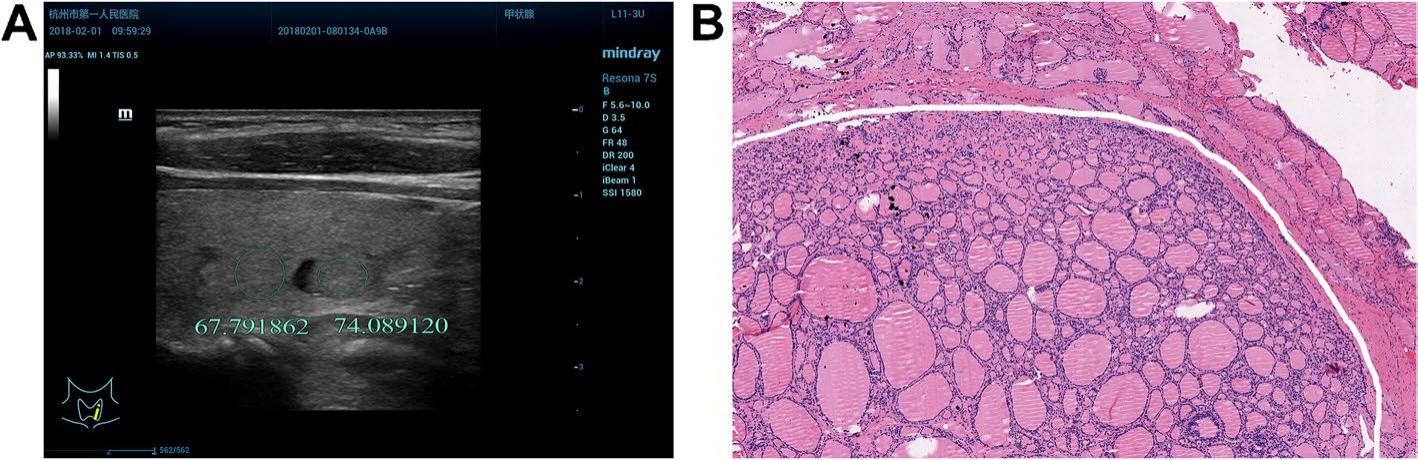
A 54-year-old woman with BMN in the left lobe of the thyroid. **A** the measurement is performed after avoiding the anechoic cystic region. The ultrasound longitudinal image shows that UGSR is 1.09 (74.09/67.79); **B** the pathological image (H&E × 40 magnification) indicates the presence of nodular goiter. The lesion is at the lower part of the image, which is separated from the normal thyroid tissues by fine fibers. Almost no lymphocyte infiltration is found within and around the lesion

**Fig. 4:**
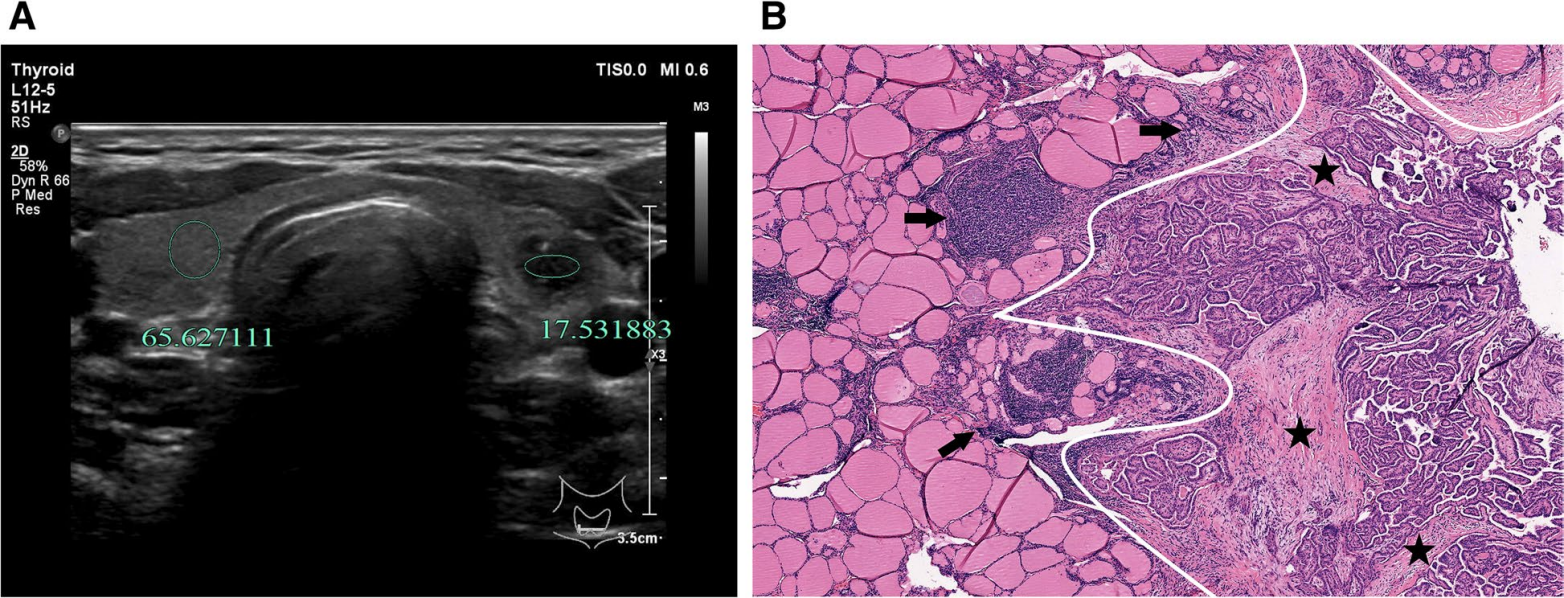
A 33-year-old woman with HT and PTMC in the left lobe of the thyroid. **A** calcification with hyperecho intensity is avoided during the measurement, and the ultrasound transverse image shows that UGSR is 0.27 (17.53/65.63). **B** the pathological image (H&E × 40 magnification) indicates the presence of HT accompanied with PTMC. The right part of the image corresponds to the PTMC, in which local fiber hyperplasia and moderate amount of lymphocyte infiltration are observed (black stars), and high amount of lymphocytes infiltration (black arrows) and lymphoid follicles formation are found around the lesion

**Fig. 5:**
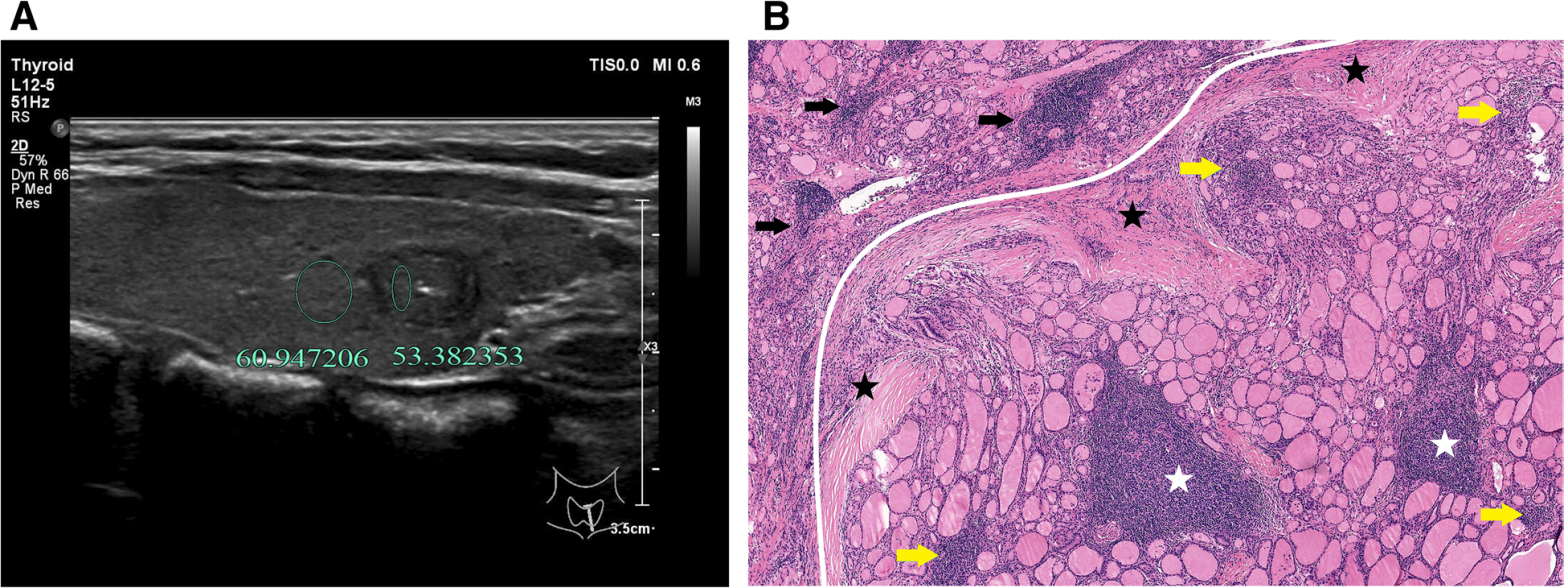
A 30-year-old woman with HT and BMN in the left lobe of the thyroid. **A** calcification with hyperecho intensity is avoided during the measurement. The ultrasound longitudinal image shows that UGSR is 0.88 (53.38/60.95). **B** the pathological image (H&E × 40 magnification) demonstrates the presence of accompanied with nodular goiter. The right part of the image corresponds to the nodular goiter, in which high amount of lymphocyte infiltration (yellow arrows) and lymphoid follicles formation (white stars) are observed. The lesion is separated from the surrounding tissues by relatively thick fibers, which are infiltrated with moderate amount of lymphocytes (black stars). Deep infiltration of the fibers is noted in the lesion, which is surrounded with high amount of lymphocyte infiltration (black arrows)

**Fig. 6:**
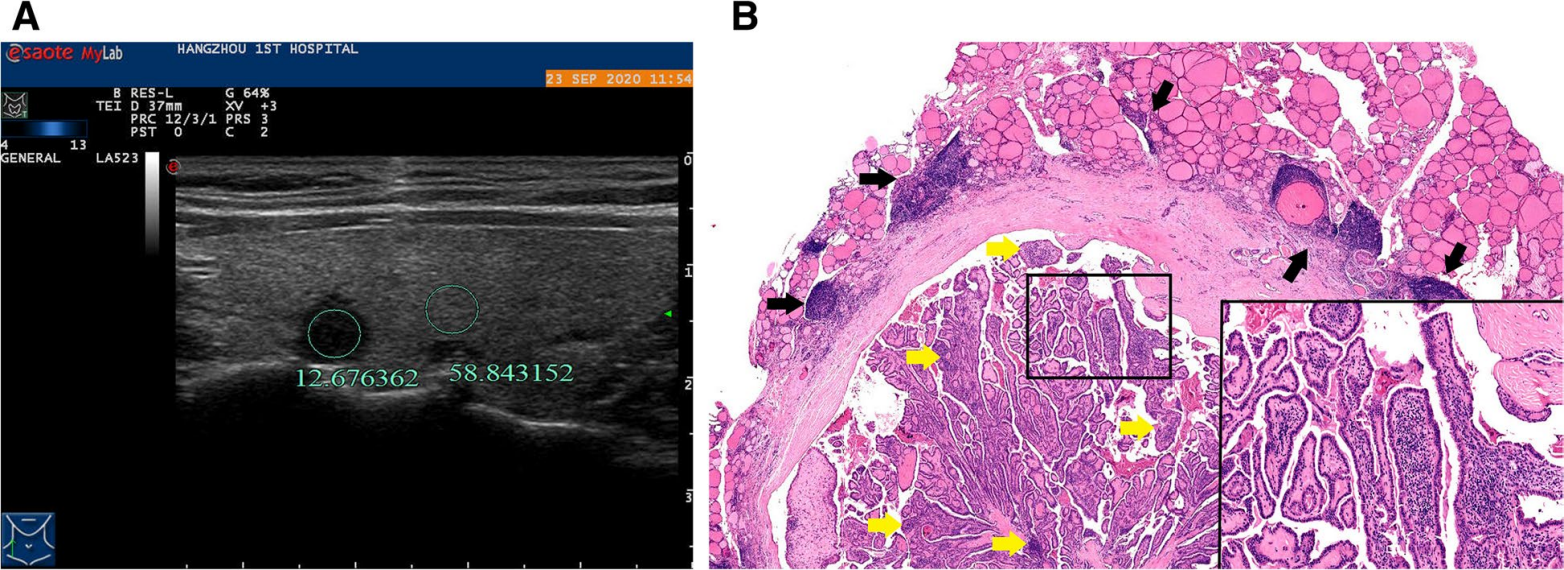
A 49-year-old woman with HT and PTMC in the right lobe of the thyroid. **A** the hyperecho strap resulting from technical factors is avoided (white arrow). The ultrasound longitudinal image shows that UGSR is 0.22 (12.68/58.84). **B** the pathological image (H&E × 10 magnification) indicates the presence of HT accompanied with PTMC. PTMC is localized at the lower part of the image and depicted in the black square at the right lower part of the image (H&E × 100 magnification). The lesion is separated from the surrounding thyroid tissues by thick fibers and collagens, and high amount of lymphocyte infiltration (yellow arrows) is noted in the lesion. The magnified image at the right lower part indicates high amount lymphocyte infiltration in the central axis of the papilla and around the lesion (black arrows)

**Fig. 7:**
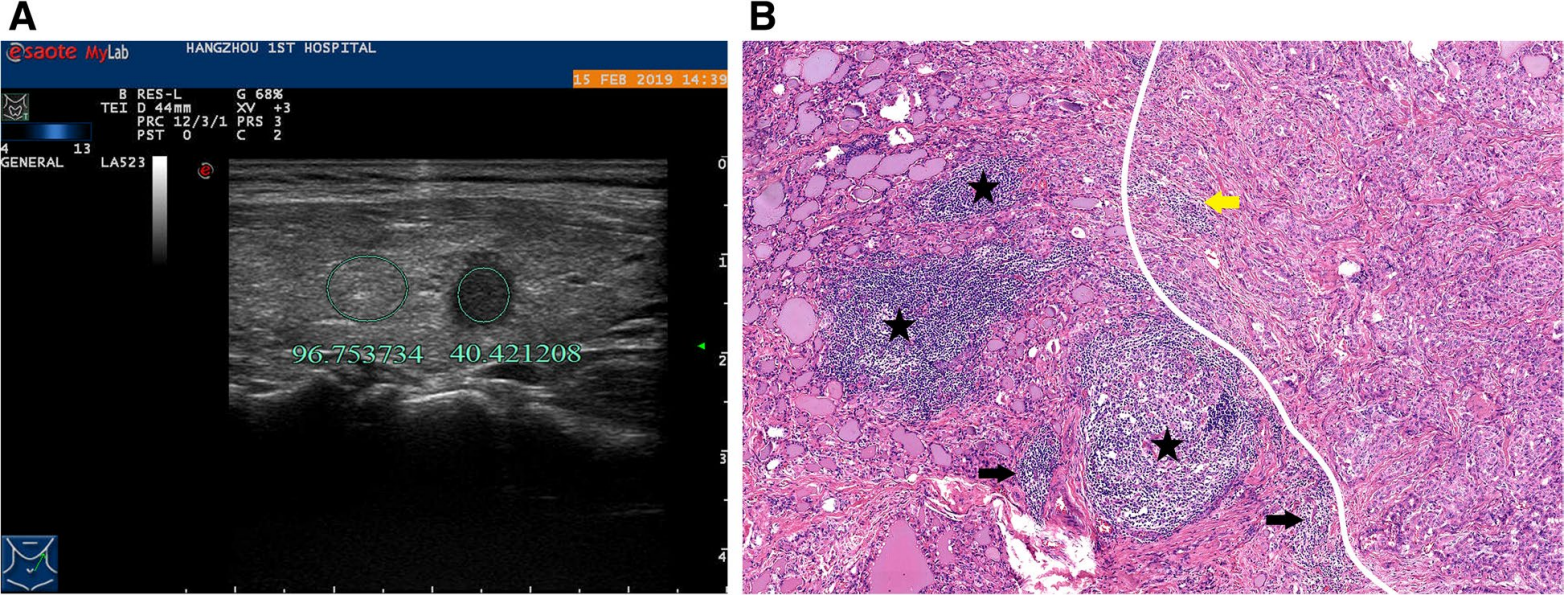
A 37-year-old woman with HT and PTMC in the left lobe of the thyroid. 6A. The hyperecho strap (white arrow) resulting from technical factors is avoided. The ultrasound longitudinal image shows that the UGSR is 0.42 (40.42/96.75). 6B, The pathological image (H&E × 40 magnification) indicates the presence of HT accompanied with PTMC. PTMC is at the right part of the image. Low amount of lymphocyte infiltration and fiber hyperplasia are found in the lesion (yellow arrow), and high amount lymphocyte infiltration and lymphoid follicles formation are found around the lesion (black stars)

A radiologist with 19 years’ working experience who were blinded to the pathological results independently analyzed the selected cases to determine the location and size of the region of interest (ROI) of the thyroid nodule and surrounding thyroid tissue. Location and size of ROI was directly related to the measurement results. The gray scale values of the nodule and surrounding thyroid tissue were measured using the gray histogram software from the RAD info reading system (Zhejiang RAD Information Technology Co., Ltd., China). The same area of the contralateral side was selected for the measurement in case of insufficient surrounding thyroid tissues on the transverse images. The larger the ROI was measured, the better the representativeness of the data for the overall echo intensity of the surrounding thyroid tissue would be. Therefore, ROI equal to or bigger than the nodule was selected in the present study to measure the surrounding thyroid tissues, which were different from the previous studies, where the ROIs with equal sizes were selected (8–10). For nodules with homogeneous echoes, the largest ROI was selected (Figs. 2, 3). For nodules with heterogenous echoes and dominated by a certain echo-dominated region, the selected ROI should be as large as possible in this region (Fig. 4, 5). For nodules with mixed echoes and when no echo-dominated region could be identified, the selected ROI should be as large as possible [8–10]. Calcifications and cystic degeneration were avoided when selecting the ROI of the nodules (Figs. 4, 5). When measuring the gray scale of the surrounding thyroid tissues, the non-nodular echo region with the highest percentage in the scanning section and with relatively homogeneous background echo was selected in the HT group (Figs. 5, 6, 7), avoiding the abnormal echo regions resulting from technical factors (Figs. 6, 7). The centers of ROI in the nodule and thyroid tissue were preferably located at the same gain level. ROIs were measured twice in all cases and the UGSR was calculated. The average value of the two measurements was designated as the UGSR of the nodule.

### Statistical analysis

Statistical analysis was performed using SPSS 21.0 (IBM Corporation, Armonk, NY, USA) and MedCalc 16.8 (MedCalc, Ostend, Belgium). Continuous variables were reported as mean ± standard deviation (SD) or median (interquartile range), as appropriate. Categorical data were reported as numbers (%). Comparison of categorical variables between the two groups was performed using the Pearson’s chi-squared test or the Fisher’s exact test, while continuous variables were compared using the independent sample t-test or the Mann–Whitney U test. Receiver operating characteristic (ROC) curve analysis was performed to explore the efficiency of UGSR in differentiating PTMCs from BMNs in the HT and non-HT groups. The area under the curve (AUC), sensitivity and specificity of UGSR in diagnosing PTMC in the two groups were compared. A *P*-value ≤ 0.05 was considered statistically significant.

## Results

### Distribution of sex, age, TPO-Ab and TG-Ab

Sex, age, TPO-Ab, and TG-Ab levels of patients in the HT group and non-HT group are shown in Table 1. There was no significant difference in sex distribution between PTMC and BMN in the HT group (*P* = 0.376) and non-HT group (*P* = 0.239). The proportion of females with PTMC and BMN were also significantly higher in the HT group than those in the non-HT group (both *P* < 0.001). In the two groups, the mean age of patients with PTMC was both significantly lower than that of patients with BMN (both *P* < 0.001). The age of patients with PTMC (*P* = 0.082) and BMN (*P* = 0.153) was not significantly different between the HT and non-HT group. In the HT group, there was no difference in TPO-Ab (*P* = 0.394) and TG-Ab (*P* = 0.073) between PTMC and BMN. In the non-HT group, there was no significant difference in TPB-Ab between PTMC and BMN (*P* = 0.108), but there was significant difference in TG-Ab between PTMC and BMN (*P* = 0.010).

**Table 1:** Distribution of sex, age, TPO-Ab, TG-Ab, nodule size and UGSR

Variable	HT group		Non-HT group	
	PTMC	BMN	PTMC	BMN
Sex, N (%)				
Male	14(5.67)	12(8.57)	125 (29.98)	50 (25.38)
Female	233 (94.33)	128 (91.43)	292 (70.02)	147(74.62)
Age, years	45.60(37.00, 54.00)	52.40(47.25, 59.00)	47.20(39.00, 56.00)	53.60(47.50, 62.00)
TPO-Ab	174.55(43.35, 1300.00)	186.35(32.90, 1300.00)	28.40(28.00, 39.00)	29.50(28.00, 40.65)
TG-Ab	119.30(53.33, 249.40)	138.35(76.83, 289.48)	25.00(17.60, 33.85)	21.20(15.00, 31.33)
Nodule size, mm	6.10 (4.00, 7.00)	6.95 (5.00, 9.00)	6.16 (5.00, 7.00)	7.08 (5.00, 9.00)
UGSR	0.52 ± 0.12	0.85 ± 0.24	0.57 ± 0.13	0.87 ± 0.20

Values are expressed as number (%), mean ± standard deviation, or median (interquartile range)

*HT* Hashimoto's Thyroiditis, *PTMC* papillary thyroid microcarcinoma, *BMN* benign micronodule, *UGSR* ultrasound gray scale ratio

### Distribution of nodule size

The maximum diameters of PTMCs and BMNs in the two groups are shown in Table 1. The maximum diameter of PTMCs was significantly smaller than that of BMNs in the HT group (*P* < 0.001) and the non-HT group (*P* < 0.001). The maximum diameter of either PTMCs (*P* = 0.688) or BMNs (*P* = 0.523) was not significantly different between the HT and non-HT group.

### Distribution of UGSR

The UGSRs of PTMCs and BMNs in the two groups are shown in Table 1.The UGSR of PTMCs was significantly lower than that of BMNs in the HT group (*P* < 0.001; Figs. 4, 5, 6, 7) and the non-HT group (*P* < 0.001; Figs. 2, 3). The UGSR of PTMC was significantly lower in the HT group than that in the non-HT group (*P* < 0.001), while the UGSR of BMN was not significantly different between the two groups (*P* = 0.416).

### Diagnostic efficiency of UGSR for PTMC and BMNs

**Fig. 8:**
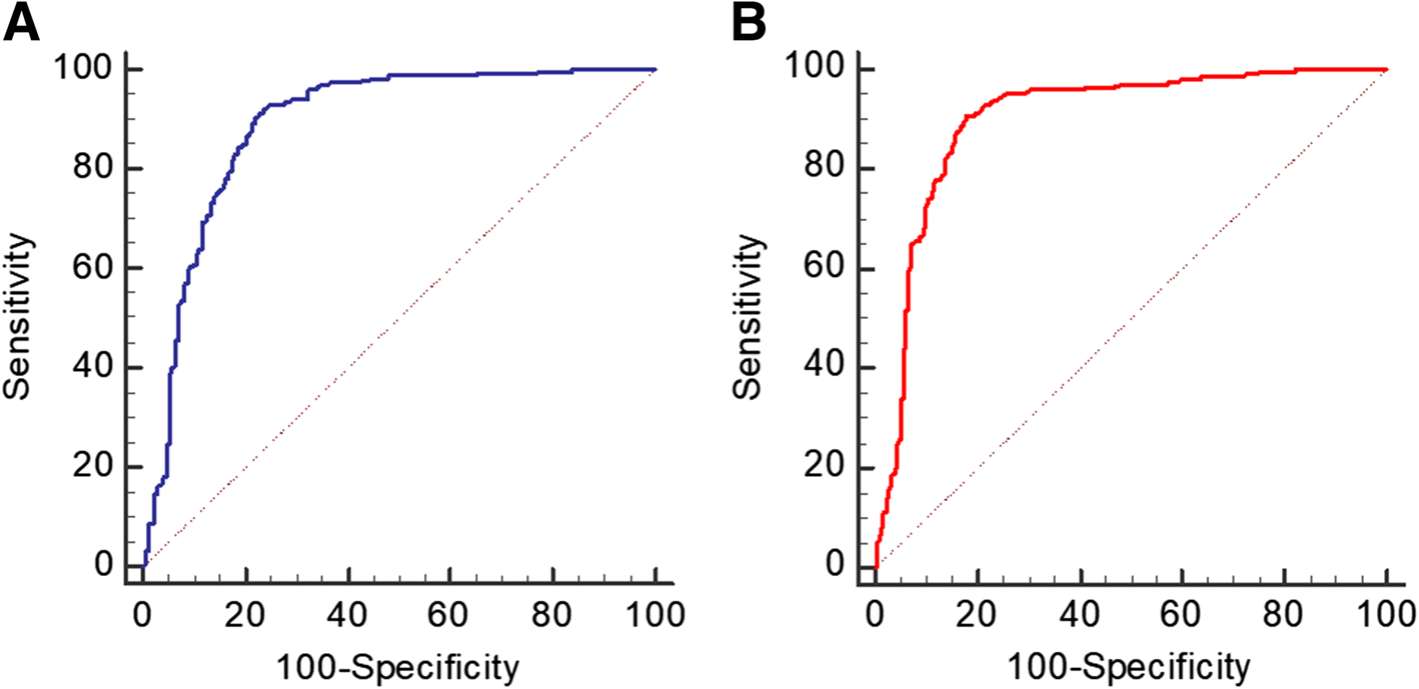
ROC curves of UGSR for the differential diagnosis of PTMC from BMN in the two groups. **A** ROC curve for the HT group. **B** ROC curve for the non-HT group

ROC curves of UGSR for differentiating PTMC from BMN in the two groups are shown in Fig. 8. AUC, optimal UGSR threshold, sensitivity and specificity were 0.890, 0.68, 91.23% and 77.46% in the HT group, and 0.901, 0.72, 90.67% and 82.05% in the non-HT group, respectively (Table 2).

**Table 2:** Diagnostic efficiency of UGSR in the HT and non-HT groups

	AUC	Optimal threshold	Sensitivity(%)	Specificity(%)
HT	0.890	0.68	91.23	77.46
non-HT	0.901	0.72	90.67	82.05

*AUC* area under the curve

### Pathological findings

Light microscopy images indicated extremely low amount or no lymphocyte infiltration inside and along the margins of PTMC and BMN in the non-HT group (Figs. 2, 3). In contrast to these observations, PTMC and BMN had a small to large amount of lymphocyte infiltration inside the margins and a large amount of lymphocyte infiltration along the margins in the HT group (Figs. 4, 5, 6, 7). Additionally, the results showed that 149 cases in the non-HT group and 81 cases in the HT group had central group lymph node metastasis.

## Discussion

HT is the most common autoimmune thyroid disease and the major cause of hypothyroidism. The incidence rate of HT in women is 4–10 times higher than that in men [16, 17]. The disease mainly occurs in patients aged 30–60 years [12, 16–19]. Positive TPO-Ab is found in 90% – 95% of patients and positive TG-Ab is observed in 60% – 80% of patients [12, 16, 17]. In the present study, tHT incidence in females was significantly higher than that in males in both groups. The incidence in females was also significantly higher in the HT group than that in the non-HT group, which was higher than previously reported results [16, 17, 20]. The age of the patients with PTMC was lower than that of patients with BMN in both groups, which could be associated with the fact that patients with malignant nodules tended to receive surgical treatment earlier. Besides, our study indicated that the TG-Ab level of BMN was lower than that of PTMC in the non-HT group. We speculated that such difference could be associated with the selection of the samples. Consistent with the previous findings [10], the average size of the PTMCs in both groups was significantly lower than that of the BMNs, which might be related to the fact that the relatively small cystic BMNs were excluded from this study for not meeting the inclusion criteria.

According to the traditional 4–5 grading method for ultrasonic echogenicity, most researchers believed that the echo intensities were of almost equal importance in differentiating benign from malignant thyroid nodules regardless of whether HT was present or not [17, 21–24], of which the sensitivity and specificity of hypoechogenicity for diagnosing malignant nodules were 62% – 87.2% and 42.9% – 58.5%, respectively [14, 15, 25]. The present study demonstrated that UGSR was statistically significant in differentiating PTMCs from BMNs in both HT and non-HT groups, and the AUC was highly consistent (0.890 and 0.901, respectively). The optimal UGSR threshold was 0.68 versus 0.72, and the corresponding sensitivity and specificity were 91.23% versus 77.46%, 90.67% versus 82.05%, respectively, suggesting that the diagnostic efficacy of the UGSR was substantially higher than that of the traditional 4–5 grading method [3, 14, 15]. The UGSR of the PTMC in the HT group was lower than that in the non-HT group, while the UGSR of the BMN was not significantly different between the two groups.

In 2015, Grani et al. [6] discriminated the thyroid nodules in patients receiving FNAC by the UGSR ratio of nodule to surrounding thyroid tissue and the UGSR ratio of nodule to neck strap muscle, demonstrating that the former had higher diagnostic power. However, in their study, the sample size of malignant nodules was small and the nodules were not classified by pathological subtype and nodule size. In addition, all nodules were confirmed by FNAC rather than histopathology. In 2018, a controlled study on UGSR of PTMC and micronodular goiters was performed in a single medical center [8], and further the team conducted a two center study in 2021 [10]. The findings of both studies demonstrated that UGSR had important value in differentiating PTMCs from micronodular goiters, and the AUC, optimal UGSR threshold, sensitivity, and specificity were highly consistent (0.895 vs. 0.918, 0.691 vs. 0.721, 86.8% vs. 88.1% and 80.4% vs. 83.3%, respectively). In 2019, Chen et al. [7] divided the papillary carcinomas and nodular goiters into three subgroups (0.3–1 cm, 1–1.5 cm and 1.5–2 cm groups) based on the size of the tumors. The results showed that as the diameter of the nodule decreased, although the specificity decreased (from 90.9% to 72.4%), the sensitivity increased significantly (from 58.8% to 97.5%). The AUC for the diagnosis of smaller-sized PTCs was the largest (0.919), which was similar to the previous findings (0.895 ~ 0.918) [8, 10]. However, these previous studies were conducted in patients with normal thyroid glands. In the present study, the AUC in the HT group, as well as the AUC and optimal UGSR threshold in the non-HT group were highly consistent with the previous studies, while the optimal UGSR threshold in the HT group was lower than that of the non-HT group and the previous findings [8, 10]. We speculated that the following two factors led to the lower UGSR in the HT group: 1) infiltration of lymphocytes resulting in decreased echogenicity of PTMC, notably in PTMCs interspersing and growing in HT background and in PTMCs with prominent lymphocyte infiltration surrounding the tumor; 2) when measuring the gray scale of thyroid gland in HT patients, the ROI contained the regions with decreased echogenicity caused by abundant lymphocyte infiltration, as well as the regions with increased echogenicity due to fibrosis. Finally, the average echo intensity of the whole ROI was close to that of the normal thyroid gland. Although the UGSR of the HT group was lower than that of the non-HT group, UGSR exhibited very high sensitivity and relatively high specificity in differentiating PTMC from BMN in the two groups, suggesting that UGSR test was also suitable for patients with HT, which was of great significance for improving the diagnostic efficiency for malignant nodules in these patients.

At present, more and more researchers advocate ultrasound-guided radiofrequency ablation or active clinical observation for low-risk PTMCs [26–29]. However, it is difficult to distinguish between low-risk and intermediate- or high-risk PTMCs clinically, especially in the judgment of lymph node metastasis. PTMC is prone to lymph node metastasis [30]. Feng et al. and Liu et al. reported that nearly one-third of central lymph node metastased in cN0 PTMC patients [31, 32]. Our study also showed that the central lymph node metastasis rate of non-HT group and HT group was 35.73% (149/417) and 32.79% (81/247), respectively. Therefore, once a low-risk PTMC could not be clearly defined, surgeons in our medical center preferred surgical resection. Besides, local patients preferred to take the initiative to require surgery due to anxiety, which was another important reason for the high operation rate of PTMC.

There were several limitations in this study. First, for nodules or thyroid tissues with heterogeneous echoes due to HT, the selection and measurement of ROI had uncertainties. In this study, the selection and measurement of the ROI was performed twice by a senior imaging specialist with 19 years’ working experience, which would greatly reduce this deviation. Second, nodular goiters, nodular HT and adenomas were included as BMN in the present study. Although we did not include all types of BMN, these three types could represent the majority of BMN [33]. Future studies will further investigate whether UGSR is also suitable for other types of BMN. Third, the judgment of the nature of thyroid nodules required the combination of multiple ultrasound features, such as morphology, taller-than-wide shape, and microcalcification, while the aim of this study was to investigate the value of UGSR for the differential diagnosis of PTMC and BMN in patients with HT. Thus, combination of UGSR with other ultrasound signs for identifying benign and malignant thyroid nodules needs to be studied further. Fourthly, this study did not present a comparison of results between different ultrasonic scanners. The main reason was that the data of this study were mainly derived from three Esaote ultrasound systems, with other models accounting for less than 20%. In the future, we will combine multi-center large sample size studies to compare UGSR obtained using different scanners. Fifthly, image analysis was performed only by one radiologist expert, and the adoption of ROIs with different sizes and shapes might reduce the reproducibility of the results. However, we chose the possibly largest ROI and measured it twice to calculate the average value, which might help to reduce this bias to some extent. Finally, the present study adopted a single-center retrospective design, while prospective studies at multiple medical centers were required to provide additional objective evidence on the diagnostic value and robustness of UGSR application in the two groups.

## Conclusions

In conclusion, UGSR had an important value in the differential diagnosis of PTMC from BMN, and the optimal UGSR threshold in the HT group was lower than that in the non-HT group. Sufficient understanding of these characteristics can improve preoperative diagnostic accuracy and provide important evidence for clinicians in selecting individualized treatment strategies.

## Data Availability

All data generated or analyzed during this study are included in this article.

## Abbreviations

UGSR: Ultrasound gray scale ratio
PTMC: Papillary thyroid microcarcinoma
HT: Hashimoto’s thyroiditis
BMN: Benign micronodule
AUC: Area under the curve
TPO-Ab: Thyroid peroxidase antibody
TG-Ab: Thyroglobulin antibody
ROI: Region of interest
ROC: Receiver operating characteristic
